# Use of antibiotics to treat humans and animals in Uganda: a cross-sectional survey of households and farmers in rural, urban and peri-urban settings

**DOI:** 10.1093/jacamr/dlaa082

**Published:** 2020-10-16

**Authors:** Susan Nayiga, Miriam Kayendeke, Christine Nabirye, Laurie Denyer Willis, Clare I R Chandler, Sarah G Staedke

**Affiliations:** 1 Infectious Diseases Research Collaboration, 2C Nakasero Hill Road, Kampala, Uganda; 2 Department of Global Health and Development, London School of Hygiene & Tropical Medicine, 15-17 Tavistock Place, London WC1H 9SH, UK; 3 Department of Politics and International Studies, University of Cambridge, The Alison Richard Building, 7 West Road, Cambridge CB3 9DT, UK; 4 Department of Clinical Research, London School of Hygiene & Tropical Medicine, Keppel Street, London WC1E 7HT, UK

## Abstract

**Background:**

Use of antibiotics to treat humans and animals is increasing worldwide, but evidence from low- and middle-income countries (LMICs) is limited. We conducted cross-sectional surveys in households and farms in Uganda to assess patterns of antibiotic use among humans and animals.

**Methods:**

Between May and December 2018, a convenience sample of 100 households in Nagongera (rural), 174 households in Namuwongo (urban) and 115 poultry and piggery farms in Wakiso (peri-urban) were selected and enrolled. Using the ‘drug bag’ method, participants identified antibiotics they used frequently and the sources of these medicines. Prevalence outcomes were compared between different sites using prevalence ratios (PRs) and chi-squared tests.

**Results:**

Nearly all respondents in Nagongera and Namuwongo reported using antibiotics to treat household members, most within the past month (74.7% Nagongera versus 68.8% Namuwongo, *P *=* *0.33). Use of metronidazole was significantly more common in Namuwongo than in Nagongera (73.6% versus 40.0%, PR 0.54, 95% CI: 0.42–0.70, *P *<* *0.001), while the opposite was true for amoxicillin (33.3% versus 58.0%, PR 1.74, 95% CI: 1.33–2.28, *P *<* *0.001).Veterinary use of antibiotics within the past month was much higher in Wakiso than in Nagongera (71.3% versus 15.0%, *P *<* *0.001). At both sites, oxytetracycline hydrochloride was the most frequently used veterinary antibiotic, but it was used more commonly in Wakiso than in Nagongera (76.5% versus 31.0%, PR 0.41, 95% CI: 0.30–0.55, *P *<* *0.001).

**Conclusions:**

Antibiotics are used differently across Uganda. Further research is needed to understand why antibiotics are relied upon in different ways in different contexts. Efforts to optimize antibiotic use should be tailored to specific settings.

## Introduction

Increased use of antimicrobial medicines for treatment of humans and animals is understood to be driving the development of antimicrobial resistance (AMR) worldwide.^1–3^ As concerns about AMR grow, global health actors have emphasized the need to reduce unnecessary use of antimicrobials.^4–6^ The WHO Global Action Plan on AMR highlights the importance of optimal use of antimicrobial medicines in human and animal health, specifically aiming to reduce consumption of antimicrobial agents worldwide.^4^ However, while evidence suggests an increasing trend in global consumption of antimicrobials, particularly antibiotics, among both humans and animals,^7^^,^^8^country-level data on antibiotic use are patchy.^9^ Current information on antibiotic consumption is drawn from import and sales data at a national level. Low- and middle-income countries (LMICs) are reportedly major contributors to the global increase in antibiotic use,^7^^,^^10^ but evidence from LMICs is limited.^11^ Data are available from only 16 African countries^7^^,^^10^ and patterns of antibiotic use in LMICs are not well understood.^11^

Evidence on hospital prescribing practices has informed stewardship programmes aiming to reduce antibiotic use in hospitals.^12–15^ However, while antibiotic use outside of hospitals is substantial,^16^^,^^17^ relatively little is known about community-level use of antibiotics in LMICs, including the geographic distribution of antibiotic use amongst households and farms, and the frequency and types of antibiotics used. Without this detailed information, interventions to optimize antibiotic use will be limited to a generic design, which has hampered the effectiveness of rational drug-use programmes in the past.^18–20^ In Uganda, the prevalence of antibiotic use in the community has been reported to range from 39% to 44%, including 43% for use of antibiotics among children <5 years of age with acute respiratory infections in the prior month in urban Kampala,^21^ 44% for treatment of cough with co-trimoxazole among children <5 years of age in the 2 weeks prior in rural Tororo,^22^ 39% for antibiotic use in the prior month among hospitalized patients in urban Kampala^23^ and 39% for antibiotic use among households reporting acute illness 2 weeks prior to the survey.^24^ Frequent use of antibiotics in poultry farms was reported by 97% of farmers in a study conducted in peri-urban Wakiso^25^ and the use of tetracycline for routine management of animal health was reported by 66% of farmers interviewed in rural Nakaseke.^26^ To better understand patterns of antibiotic use in Uganda, we conducted cross-sectional surveys in three geographic locations, focusing on both households and piggery and poultry farms where antibiotic use has been reported, but is not yet well described.^21^^,^^22^^,^^25^

## Methods

### Study sites

**Figure 1.**
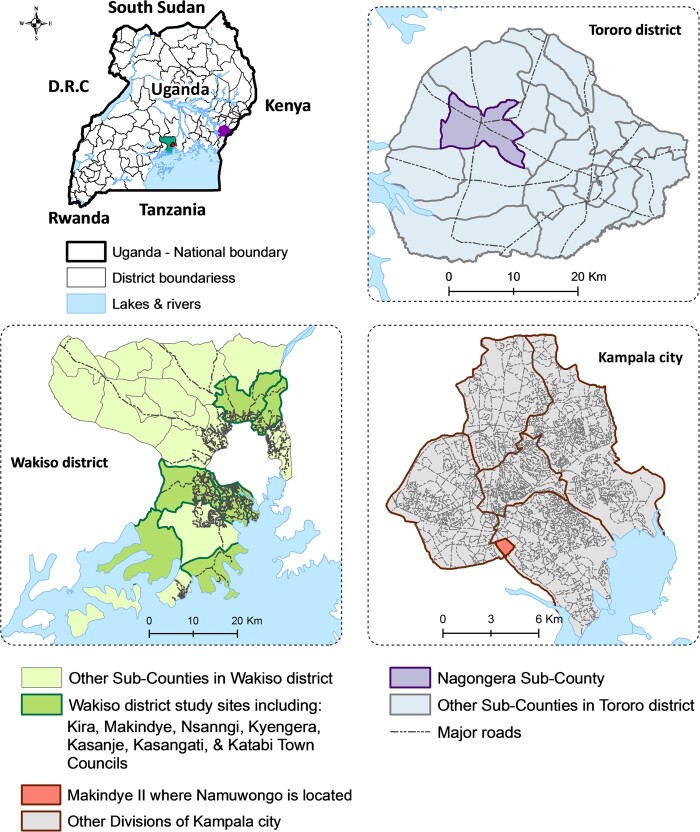
Map of study areas. The study was conducted in: (1) Nagongera subcounty, Tororo district; (2) Namuwongo informal settlement, Kampala city; (3) Kira, Makindye, Nsangi, Kyengera, Kasanje, Kasangati and Kabati town councils, Wakiso district.

Cross-sectional surveys were conducted in the following three locations (Figure 1). (1) Nagongera subcounty is in Tororo district in Eastern Uganda, a rural area where most residents engage in agriculture as their main economic activity.^27^ In Nagongera, we collected information on antibiotic use for humans and any animals associated with the households. (2) Namuwongo, in Kampala city, is a large informal settlement where many people who work in the city centre and the surrounding affluent neighbourhoods reside.^28^ In Namuwongo, where few animals are kept, we collected data on antibiotic use among humans only. (3) Wakiso district is a peri-urban area approximately 20 km north-west of Kampala and is an agricultural district that has been ranked as a top producer of poultry and piggery in Uganda.^29^ In Wakiso, we collected data on antibiotic use from small- and large-scale poultry and piggery farmers.

### Recruitment

Potential participants were identified with the help of field guides, including local council leaders and village health team members in Nagongera and Namuwongo, and animal health workers in Wakiso. In Nagongera and Namuwongo, households were included if: (1) at least one adult (≥18 years old) was present; (2) household members were permanent residents (lived in the area for at least 6 months); and (3) the adult agreed to provide written informed consent. In Wakiso, farms were included if the farm owner: (1) was present or could be reached by phone; and (2) agreed to provide written informed consent. Households and farms were excluded if an adult resident or farm owner could not be located after at least two visits. Participants were selected using convenience sampling, but we attempted to recruit a cross-sectional sample of the population in each study site.

### Survey procedures

Prior to the surveys, the study team met with local health and veterinary officials, and village leaders, to discuss the survey plans. The survey was conducted using the ‘drug bag’ method.^30^ First, we visited local drug shops, pharmacies, private clinics and public health facilities. With the help of Ugandan pharmacists, we compiled a list of antibiotics available for human and animal use. Subsequently, we purchased packets, bottles, tablets, capsules and vials of the antibiotics reported to be most commonly requested. These medicines were put into drug bags, one for human antibiotics and another for animal antibiotics. During the surveys, we presented the drug bag to participants and asked them to ‘pile sort’ the medicines into four different categories, including drugs they: (1) recognized; (2) had ever used; (3) used frequently; and (4) needed, but could not get. While the participants sorted the medicines, we used a pre-set questionnaire to gather information about their experiences using these medicines.

### Data management and statistical analysis

Data were collected using hand-held tablets, which were programmed using Open Data Kit (ODK) (accessible at www.opendatakit.org). We classified antibiotic use into two categories: (1) any antibiotic use (ever used): defined as taking any antibiotic, for any indication, with any dosage, with or without a prescription, as reported by participants; and (2) frequent antibiotic use: self-reported by study participants during the pile-sorting exercise and defined during analysis as antibiotic use within the past month. Questionnaire data were transferred daily from the tablets to a password-protected laptop. At the end of the survey the complete databases were stored on a secure server at the Infectious Diseases Research Collaboration (IDRC) in Kampala. Data were analysed using Stata 14 (StataCorp LLC, College Station, TX, USA).

Antibiotics for human use were categorized using the WHO AWaRe classification^31^ as: (1) Access: first- and second-choice antibiotics for common infections that should be widely available, affordable and quality assured; (2) Watch: first- and second-choice antibiotics recommended for a specific and limited number of indications, given their association with AMR; and (3) Reserve: antibiotics that should be treated as a ‘last resort’, limited to highly specific patients and settings, and used only when all alternatives have failed. The WHO AWaRe classification aims to inform effective antimicrobial stewardship and ensure access to necessary antibiotics and appropriate prescribing.^31^ Antibiotics used for animals were interpreted using the WHO list of critically important antimicrobials for human medicine (WHO CIA), which classifies antimicrobials as: (1) critically important; (2) highly important; or (3) important, based on their indications for treatment of humans. This list aims to ensure that antimicrobials, particularly those classified as critically important, are used with caution, both in human and veterinary medicine.^32^

In the analysis, descriptive statistics were generated and proportions were reported for each variable. Chi-squared tests were used to compare participant characteristics between the study sites. Prevalence ratios (PRs) were generated for comparisons between the study sites of data on prevalence of any antibiotic use, frequent antibiotic use, different antibiotics used and the source of antibiotics. The PR in our study was the ratio of the outcome of interest (proportion of participants who reported any antibiotic use, frequent antibiotic use, use of different antibiotics and source of antibiotics) divided by the proportion of participants surveyed, in a given geographic location.

### Ethics

We obtained ethics approval for the study from the School of Biomedical Sciences Research and Ethics Committee, Makerere University College of Health of Sciences (SBSREC REF no. 562), the Uganda National Council for Science and Technology (SS 4679) and the London School of Hygiene and Tropical Medicine Ethics Committee (LSHTM Ethics Ref: 15244).

## Results

### Baseline characteristics

**Figure 2.**
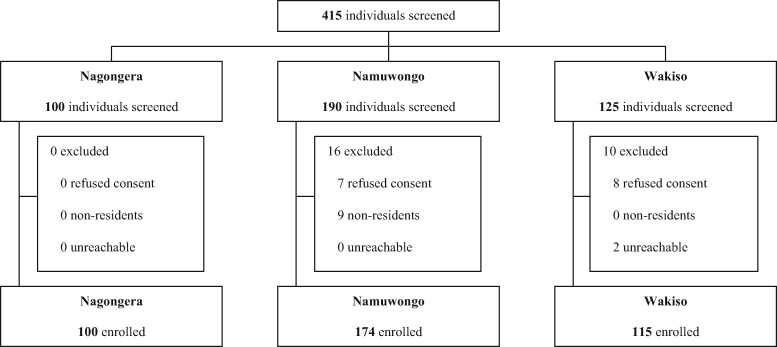
Trial profile. Outlining the process of recruitment, screening and enrolment into the study. In total, 100 participants were enrolled in Nagongera, 174 in Namuwongo and 115 in Wakiso.

From November to December 2018, 100 participants were enrolled in Nagongera, and from May to June 2018, 174 and 115 participants were enrolled in Namuwongo and Wakiso, respectively (Figure 2). The characteristics of participants enrolled in the three sites varied (Table 1). Considering the population evaluated for antibiotic use in humans, more respondents were female in Namuwongo than in Nagongera (79.3% versus 56.0%, respectively). In Nagongera, 89.0% of respondents were subsistence farmers, while in Namuwongo, 85.1% were either merchants or labourers (involved in making, building or fixing things, or cooking and cleaning). Considering the population evaluated for antibiotic use in animals, respondents in Nagongera and Wakiso were similar in gender and age (Table 1), but education, occupation and farm characteristics varied. In Nagongera, 16.0% of respondents had received no education and 49.0% only reached primary school, while in Wakiso, 78.8% of participants reached secondary school or higher. In Nagongera, no respondents owned their farm, while in Wakiso, 72.2% were farm owners. In Nagongera, all farms were classified as subsistence farms with little capacity to produce surplus for the market, while all farms in Wakiso were commercial, mostly small-scale farms.

**Table 1.** Participant characteristics

Human use	Nagongera	Namuwongo	*P* value
Setting	rural	urban	
Sample size	100	174	
Gender of respondent, female	56 (56.0%)	138 (79.3%)	<0.001
Occupation^a^			
Subsistence farmer	89 (89.0%)	7 (4.0%)	<0.001
Merchant	1 (1.0%)	103 (59.2%)	
Labourer	1 (1.0%)	45 (25.9%)	
Other	9 (9.0%)	19 (10.9%)	

Animal use	Nagongera	Wakiso	*P* value

Setting	rural	peri-urban	
Sample size	100	115	
Gender of respondent, female	56 (56.0%)	55 (47.8%)	0.23
Age (years)			
<40	39 (39.0%)	41 (35.7%)	0.61
≥40	61 (61.0%)	74 (64.3%)	
Highest level of education^b^			
Never went to school	16 (16.0%)	1 (0.9%)	<0.001
Primary	49 (49.0%)	23 (20.4%)	
Secondary or higher	35 (35.0%)	89 (78.8%)	
Occupation^c^			
Subsistence farmer	89 (89.0%)	0	<0.001
Farm owner	0	83 (72.2%)	
Farm worker	0	32 (27.8%)	
Other	11 (11.0%)	0	
Farm categories^d^			
Poultry			
Subsistence	85 (85%)	0	<0.001
Small (<5000 birds)	0	61 (95.3%)	
Large (≥5000 birds)	0	3 (4.7%)	
Piggery			
Subsistence	38 (38%)	0	<0.001
Small (<30 pigs)	0	47 (72.3%)	
Large (≥30 pigs)	0	18 (27.7%)	

a Merchant: engaged in selling food, drinks and other items; labourer: making, building, fixing, cooking, cleaning; other: students (*n *=* *2), witch doctors (*n *=* *1), drivers (*n *=* *2), boda drivers (*n *=* *1), businessmen (*n *=* *1), rent collectors (*n *=* *1), teachers (*n *=* *4), security guards (*n *=* *8), factory workers (*n *=* *5), masons (*n *=* *1), unemployed (*n *=* *2).

b In Wakiso, two respondents refused to answer and were excluded (*n *=* *113); secondary or higher: secondary-level education, certificate, diploma, vocational training and university degree.

c Farm worker: anyone employed at the piggery or poultry farm, including farm managers and other workers; other: teachers (*n *=* *4), students (*n *=* *2), businessmen (*n *=* *1), masons (*n *=* *1), food sellers (*n *=* *1), unemployed (*n *=* *2).

d In Wakiso, poultry farms *n *=* *64, piggery farms *n *=* *65; farms defined using the Food and Agricultural Organisation 2014 criteria. Subsistence farms: those that produce for the farmer’s own consumption and with little or no capacity to generate surplus production for the market; small farms: those that are either market-oriented and commercial, generating surplus production for a market (local, national or international), or have the potential to become market-oriented; large farms: those showing characteristics of industrial ventures.

### Patterns and sources of antibiotics in humans

**Figure 3.**
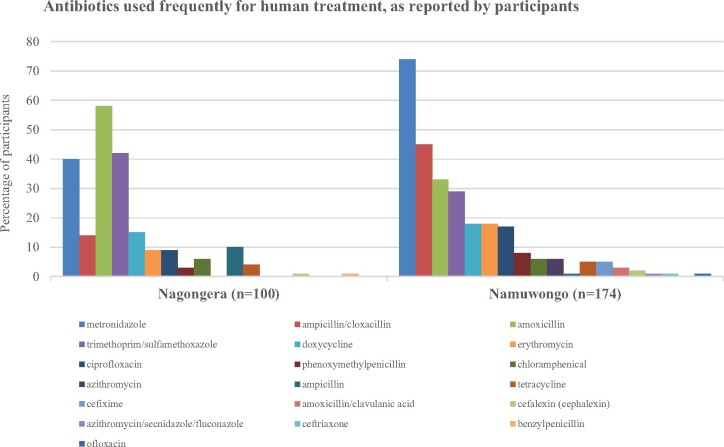
Antibiotics used frequently for human treatment, as reported by participants. Antibiotics were identified by participants using the drug bag method and are presented as the percentage of participants who reported using the antibiotic frequently to treat members of their household. In total, 18 antibiotics in Namuwongo and 13 in Nagongera were identified.

Nearly all respondents in Nagongera and Namuwongo reported using antibiotics to treat illness in their households (Table 2), most within the past month (74.7% in Nagongera versus 68.8% in Namuwongo). Far more participants in Nagongera reported obtaining medicines from public health facilities than in Namuwongo (84.2% versus 22.9%), but in both areas most participants reported obtaining medicines from the private sector (87.4% in Nagongera versus 75.9% in Namuwongo). In Namuwongo, the drug bag contained 24 antibiotics; 21 of these were recognized by respondents and 18 were used frequently (Figure 3). In Nagongera, the drug bag contained only 20 antibiotics; 16 of these were recognized by respondents and 13 were used frequently. In both sites, the drug bag for humans did not include any antibiotics classified as ‘reserve’ drugs because these drugs were rarely requested. The most frequently used antibiotic in Nagongera was amoxicillin, while in Namuwongo it was metronidazole (Table 3). Use of amoxicillin was significantly more common in Nagongera than in Namuwongo (58.0% versus 33.3%, PR 1.74, 95% CI: 1.33–2.28, *P *<* *0.001), while the opposite was true for metronidazole (40.0% versus 73.6%, PR 0.54, 95% CI: 0.42–0.70, *P *<* *0.001). Ampicillin/cloxacillin and trimethoprim/sulfamethoxazole were also frequently used, ampicillin/cloxacillin more commonly in Namuwongo (45.4% in Namuwongo versus 14.0% in Nagongera) and trimethoprim/sulfamethoxazole more commonly in Nagongera (42.0% in Nagongera versus 28.7% in Namuwongo). Ciprofloxacin and erythromycin, classified as drugs to ‘watch’ in the WHO AWaRe system, were used more often in Namuwongo than in Nagongera (ciprofloxacin PR 0.54, 95% CI: 0.27–1.09, *P *=* *0.10 and erythromycin PR 0.49, 95% CI: 0.24–0.98, *P *=* *0.04), although this difference was significant only for erythromycin.

**Table 2.** Antibiotic use and the source of medicines for treatment of humans

Human use	Nagongera (*n *=* *100)	Namuwongo (*n *=* *174)	PR (95% CI)	*P* value
Ever use antibiotics^a^				
Yes	95 (95.0%)	170 (97.7%)	0.97 (0.92–1.02)	0.29
No	5 (5.0%)	4 (2.3%)		
Frequency of antibiotic use^b^				
≤1 month	71 (74.7%)	117 (68.8%)	1.09 (0.93–1.27)	0.33
>1 month	24 (25.3%)	53 (31.2%)	0.81 (0.54–1.22)	0.33
Source of medicines				
Public health facilities	80 (84.2%)	39 (22.9%)	3.67 (2.75–4.90)	<0.001
Research/non-governmental organizations	1 (1.1%)	8 (4.7%)	0.22 (0.03–1.76)	0.16
Other^c^	83 (87.4%)	129 (75.9%)	1.15 (1.03–1.29)	0.03

a Antibiotic use was defined as taking any antibiotic for any indication at whatever dosage as reported by participants.

b In Nagongera, participants reported how often any antibiotic was used to treat any member of the household for any indication and in any dosage; in Namuwongo, participants reported the last time any antibiotic was used for any indication and in any dosage, with or without a prescription.

c Other: private clinics, pharmacies and drug shops.

**Table 3.** Antibiotics used frequently for human treatment, as reported by participants

Antibiotic classes for human use	Antibiotic	WHO classification (AWaRe)^a^	Nagongera	Namuwongo	PR (95% CI)	*P* value
Penicillin	amoxicillin	Access	58 (58.0%)	58 (33.3%)	1.74 (1.33–2.28)	<0.001
	ampicillin	Access	10 (10.0%)	1 (0.6%)	17.4 (2.26–133.93)	<0.001
	phenoxymethylpenicillin	Access	3 (3.0%)	14 (8.1%)	0.37 (0.11–1.27)	0.12
	ampicillin/cloxacillin	Access	14 (14.0%)	79 (45.4%)	0.31 (0.18–0.51)	<0.001
Cephalosporin	cefalexin	Access	1 (1.0%)	4 (2.3%)	0.44 (0.05–3.84)	0.66
Metronidazole	metronidazole	Access	40 (40.0%)	128 (73.6%)	0.54 (0.42–0.70)	<0.001
Sulphonamide	trimethoprim/ sulfamethoxazole	Access	42 (42.0%)	50 (28.7%)	1.46 (1.05–2.03)	0.03
Fluoroquinolone	ciprofloxacin	Watch	9 (9.0%)	29 (16.7%)	0.54 (0.27–1.09)	0.10
Chloramphenicol	chloramphenicol	Access	6 (6.0%)	11 (6.3%)	0.95 (0.36–2.49)	1.00
Macrolide	erythromycin	Watch	9 (9.0%)	32 (18.4%)	0.49 (0.24–0.98)	0.04
Tetracycline	tetracycline	Access	4 (4.0%)	9 (5.2%)	0.77 (0.24–2.45)	0.77
	doxycycline	Access	15 (15.0%)	31 (17.8%)	0.84 (0.48–1.48)	0.62

a The WHO’s AWaRe classification aims at informing effective antimicrobial stewardship and ensuring access to necessary antibiotics and appropriate prescribing; categories include ‘Access’, ‘Watch’ and ‘Reserve’. Access: first- and second-choice antibiotics for common infections that should be widely available, affordable and quality assured; Watch: first- and second-choice antibiotics recommended for specific and limited number of indications because they have a higher potential for development of resistance.

### Patterns and sources of antibiotics in animals

**Figure 4.**
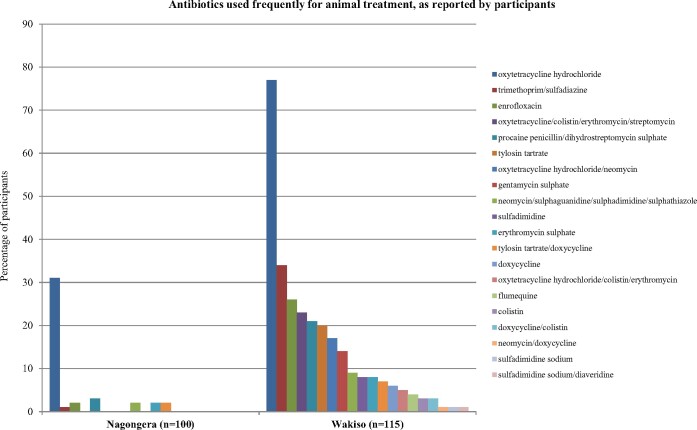
Antibiotics used frequently for animal treatment, as reported by participants. Antibiotics were identified by participants using the drug bag method and are presented as the percentage of participants who reported using the antibiotic frequently to treat animals of their household. In total, 20 antibiotics in Wakiso and 7 in Nagongera were identified.

Veterinary use of antibiotics was reported in both Wakiso and Nagongera (Table 4) but was far more common in Wakiso (86.1% versus 33.0%, respectively). Of those participants who reported ever using antibiotics to treat animals, significantly more participants in Wakiso had used antibiotics over the past month (82.8% in Wakiso versus 45.5% in Nagongera, PR 0.55, 95% CI: 0.37–0.81, *P *<* *0.001). In both areas, medicines were obtained frequently from the private sector. In Wakiso, the antibiotic bag contained 21 antibiotics; all of these were recognized by respondents and 20 were used frequently (Figure 4). In Nagongera, the drug bag contained only 16 antibiotics; 10 of these were recognized by respondents and 7 were used frequently. In both sites, all 15 of the antibiotics classified as ‘critically important’ that were included in the drug bags were recognized by participants in Wakiso, while only 7 were recognized in Nagongera. In both sites, the most frequently used antibiotic was oxytetracycline hydrochloride (Table 5), which was used more often in Wakiso than in Nagongera (76.5% versus 31.0%, respectively, PR 0.41, 95% CI: 0.30–0.55, *P *<* *0.001). In Wakiso, three other commonly used antibiotics (dihydrostreptomycin sulphate, erythromycin sulphate and tylosin tartrate) are classified as ‘critically important’ in the WHO CIA system; all three were used more often in Wakiso than in Nagongera (Figure 4, Table 5), although this difference was not significant for erythromycin sulphate. Of note, use of colistin, another ‘critically important’ antimicrobial, was reported by some respondents in Wakiso, either alone or in combination with other antibiotics.

**Table 4.** Antibiotic use and the source of medicines for treatment of animals

Animal use	Nagongera (*n *=* *100)	Wakiso (*n *=* *115)	PR (95% CI)	*P* value
Ever use antibiotics^a^				
Yes	33 (33.0%)	99 (86.1%)	0.33 (0.25–0.44)	<0.001
No	67 (67.0%)	1 (0.9%)		
Frequency of antibiotic use^b^				
≤1 month	15 (45.5%)	82 (82.8%)	0.55 (0.37–0.81)	<0.001
>1 month	18 (54.5%)	17 (17.2%)	3.18 (1.86–5.41)	<0.001
Source of medicines				
Veterinary pharmacy/drug shop	30 (90.9%)	77 (77.8%)	1.17 (1.01–1.36)	0.13
Veterinary officer	0	23 (23.2%)	0	0.001
Market	3 (9.1%)	0		0.01

a Antibiotic use was defined by participants as using any antibiotic for treatment of animals for any indication at any dose; in Wakiso, 15 respondents didn’t know if antibiotics had been used to treat their animals (*n *=* *115).

b In Nagongera, participants reported how often any antibiotic was used to treat animals kept by the household for any indication and in any dosage; in Wakiso, participants reported the last time any antibiotic was used on the farm for any indication and in any dosage.

**Table 5.** Antibiotics used frequently for treatment of animals

Antibiotic classes for animal use	Antibiotic	WHO classification (CIA)^a^	Nagongera	Wakiso	PR (95% CI)	*P* value
Penicillin/aminoglycoside	procaine penicillin/	highly important	3 (3.0%)	24 (20.9%)	0.14 (0.04–0.46)	<0.001
	dihydrostreptomycin sulphate	critically important	3 (3.0%)	24 (20.9%)	0.14 (0.04–0.46)	<0.001
Sulphonamide	trimethoprim/sulfadiazine	highly important	1 (1.0%)	39 (33.9%)	0.03 (0.004–0.21)	<0.001
Macrolide	erythromycin sulphate	critically important	2 (2.0%)	9 (7.8%)	0.26 (0.06–1.16)	0.07
	tylosin tartrate	critically important	0	23 (20.0%)	0	<0.001
Tetracycline	oxytetracycline hydrochloride	highly important	31 (31.0%)	88 (76.5%)	0.41 (0.30–0.55)	<0.001

a The WHO’s CIA classification aims at ensuring that antimicrobials, particularly those classified as critically important, are used with caution both in human and veterinary medicine; categories include (1) critically important, (2) highly important, and (3) important, based on their indications for treatment of humans.

## Discussion

Current efforts to optimize antibiotic use outside of hospitals rely on key messages to increase awareness of AMR and discourage the misuse of antibiotics. However, lack of data to inform these messages, and the generic nature of centralized global messaging, limit their impact on antibiotic use.^33^^,^^34^ Our findings provide important insights into the current status of antibiotic use in Uganda. Here, the stark difference in patterns of antibiotic use in three different settings—rural, urban and peri-urban—suggests that interventions will need to be tailored to specific sites and populations.

Overall, the frequency of antibiotic use amongst residents and farmers was high, underscoring a trend signalled in estimates of global use of antibiotics for human treatment, derived from import/sales data,^7^^,^^10^ as well as the projected trajectory of antibiotic use in animals based on increasing livestock farming.^35^ High levels of antibiotic use have been reported amongst residents in rural Nigeria where 82% of respondents had used an antibiotic in the past 6 months,^36^ and in an informal settlement in urban Kenya where 87% reported using antibiotics in the last 12 months.^37^ However, other research studies, conducted in Uganda and elsewhere in Africa between 2007 and 2017, have reported lower prevalence of antibiotic use; in a household survey conducted in five African countries (The Gambia, Ghana, Nigeria, Uganda and Kenya) in 2007–08, use of antibiotics to treat acute illness within the past 2 weeks ranged from 17.6% to 42.3% in Kenya.^24^ Similar results have been reported from urban settings across Africa; antibiotic use in the prior month was 39% among hospitalized patients in Uganda^23^ and 57% amongst residents in Nigeria,^38^ while 49% of residents in Ethiopia reported antibiotic use within the past year.^39^ Our findings of frequent antibiotic use amongst piggery and poultry farmers in Wakiso, an area with increasing commercial and semi-industrialized farms, also mirror rates of antibiotic use reported elsewhere in Africa: 100% of respondents on commercial poultry farms in Tanzania^40^ and Ogun State, Nigeria^41^ reported frequently using antibiotics, while in Abia State, Nigeria 65% of commercial poultry farms and 40% of piggery farms used an antibiotic weekly and fortnightly, respectively.^42^ Improvements in technology, changes in the global economy, rapid population growth and increased consumption of livestock products have been reported as factors that have influenced changes globally in farming, with many subsistence farms, with little capacity to produce surplus for sale on the market, transitioning to commercial and market-based farms, which are associated with routine use of antimicrobials.^35^^,^^43^ The shift away from subsistence farming towards commercial and market-based farming could explain the rates of antibiotic use observed in commercial farms in our study sites and in studies conducted elsewhere in Africa.^8^^,^^35^

The types of antibiotics used by residents and farmers varied widely between the three geographies. In Namuwongo, 74% of participants reported using metronidazole frequently, compared with only 40% of households in rural Nagongera. However, in both sites, use of metronidazole was much higher than reports from elsewhere in Africa.^24^^,^^39^ In the study conducted in five African countries, use of metronidazole in individuals with an acute illness who received antibiotics was 17.2% overall, ranging from 10% to 27%; in Uganda, only 11.3% received metronidazole.^24^ The potential impact of such high use of metronidazole, as seen in our study, on the development of AMR requires exploration. There are numerous potential mechanisms for metronidazole resistance to occur,^44–46^ but the degree to which the high rates of metronidazole use have impacted (or will impact) microbial communities, and subsequent susceptibility to therapy, remains unknown. Overuse of metronidazole may also damage beneficial gut microbial populations, which may have a negative impact on human health and increase susceptibility to infections and disease.^47^^,^^48^ The high levels of metronidazole use also require exploration with qualitative research, to understand the reasons and history of the use of this antibiotic in our study area. Use of other antibiotics by our study participants was more comparable across settings, and similar to reports from elsewhere, with quite a narrow range of ‘Access’ category antibiotics available and used frequently, notably penicillins. Further work is required to establish whether ciprofloxacin and erythromycin, drugs to ‘watch’ in the WHO AWaRe system, were prescribed, as these drugs were used less frequently in our study sites. The range of antibiotics commonly used to treat animals was wider in Wakiso than in Nagongera, primarily a subsistence farming area, but nonetheless most antibiotics were not ‘critically important’, except for dihydrostreptomycin sulphate, erythromycin sulphate and tylosin tartrate. Notably, a few participants in Wakiso reported using colistin frequently. The most commonly used antibiotic in both sites was oxytetracycline hydrochloride, consistent with findings reported from studies conducted between 1998 and 2018 on commercial farms from Tanzania,^40^ Ghana^49^ and Nigeria^50^ and a wider review of veterinary use of antimicrobials in LMICs.^51^

Antibiotics were mainly acquired through the private sector for both human and animal use in all three study areas. This is consistent with results of prior studies of antibiotic access for humans in Uganda,^52^ Ethiopia^39^ and Tanzania,^53^ for commercial poultry production in Nigeria^41^ and Ghana,^49^ and studies of antibiotic access for animals in Rwanda.^54^ Understanding the forces that draw people to the private sector is important, including how the pharmaceutical industry operates and is regulated. The favourable tax environment in Uganda, where no taxes are levied on imported pharmaceuticals for either humans or animals,^55^ and a growing pharmaceutical market valued at US$276 million in 2010^56^ and $414 million in 2017,^57^ enable the pharmaceutical industry to flourish while being dominated by imported pharmaceuticals. With the deterioration of the public health system for both humans and animals over many years,^58^^,^^59^ it has been estimated that 60%–70% of human healthcare services and all veterinary clinical services in Uganda are provided by the private sector.^58–60^ These factors enable the existence of a dominant private sector in Uganda today.

Our study had several important limitations. First, our findings are based on self-reported use of antibiotics, which may not reflect the full picture of antibiotic use for human and animal health. However, we do expect our findings to be a more accurate representation of self-reporting due to the use of physical samples with the drug bag method in both homes and farms in order to avoid linguistic and classification errors in antibiotic knowledge.^30^ Second, we relied on convenience sampling to recruit participants into the surveys. Thus, the findings from these surveys are not generalizable to the wider Ugandan population, but they do provide insights into antibiotic-use experiences from three different contexts, underscoring the uniqueness of each setting. Third, we relied on a self-determined classification of ‘frequently used’ for antibiotics, which could vary between individuals. This was in recognition of inaccuracies in reporting health events for time periods over about 2 weeks^61^ and reflected our interest in whether these medicines were often used, rather than just in a recent time period. Finally, very few large-scale farms were included, which may create a gap for further research to fully understand antibiotic-use experiences in such spaces.

The implications of these findings for policy lie in the need to recognize the increasing reliance on many of these medicines and the important role of the private sector in providing access to antibiotics.

Interventions aimed at changing the knowledge and behaviour of healthcare professionals and the general public through education on the importance of using antimicrobials appropriately, and the dangers that may arise from the misuse of antimicrobials, have had limited impact in some areas.^20^ Lessons can be learned from the challenges faced in attempting to reduce antibiotic use through knowledge and awareness programmes alone.^62^^,^^63^ Rather, an in-depth understanding of the relationships between people, animals and medicines could provide alternative paths to intervention. Further research is required to understand why we found such heterogeneity between local geographies in the frequency and types of antibiotics used, and why particular antibiotics were so commonly used. Such research should trace the histories and current everyday realities of particular antibiotics across different settings to help clarify why and how antibiotics are used in different settings. It is also essential to harmonize these results with medical microbiological data to understand the impacts that short- and longer-term trends of antibiotic use may have on microbial populations and drug-resistant infections. Finally, our findings demonstrate the ongoing importance of addressing the roles of antimicrobial markets beyond the formal health sector when developing programmes to optimize antibiotic use.
